# Transplantation of CRISPRa system engineered IL10-overexpressing bone marrow-derived mesenchymal stem cells for the treatment of myocardial infarction in diabetic mice

**DOI:** 10.1186/s13036-019-0163-6

**Published:** 2019-05-30

**Authors:** Xin Meng, Minjuan Zheng, Ming Yu, Wei Bai, Lei Zuo, Xin Bu, Yi Liu, Linying Xia, Jing Hu, Liwen Liu, Jianping Li

**Affiliations:** 1 Department of Ultrasonography, Xijing Hospital, the Fourth Military Medical University, Xi’an, 710032 Shaannxi China; 2 Department of Biochemistry and Molecular Biology, the Fourth Military Medical University, Xi’an, 710032 Shaannxi China; 3 Department of Cardiology, Xijing Hospital, the Fourth Military Medical University, Xi’an, 710032 Shaannxi China; 4 Department of Radiation Oncology, Xijing Hospital, the Fourth Military Medical University, Xi’an, 710032 Shaannxi China

**Keywords:** CRISPRa, IL-10, BM-MSCs, Myocardial infarction, Diabete

## Abstract

**Background:**

Myocardial infarction (MI) is a common cause of mortality in people. Mesenchymal stem cell (MSC) has been shown to exert therapeutic potential to treat myocardial infarction (MI). However, in patients with diabetes, the diabetic environment affected MSCs activity and could impair the efficacy of treatment. Interleukin-10 (IL-10) has been shown to attenuate MI by suppressing inflammation. In current study, the combination of MSC transplantation with IL-10 was evaluated in a diabetic mice model with MI.

**Methods:**

We engineered bone marrow derived MSCs (BM-MSCs) to overexpress IL-10 by using CRISPR activation. We established the diabetic mice model with MI and monitored the IL-10 expression after BM-MSCs transplantation. We also evaluated the effects of BM-MSCs transplantation on inflammatory response, cell apoptosis, cardiac function and angiogenesis.

**Results:**

CRISPR activation system enabled overexpression of IL-10 in BM-MSCs. Transplantation of BM-MSCs overexpressing IL-10 resulted in IL-10 expression in heart after transplantation. Transplantation of BM-MSCs overexpressing IL-10 inhibited inflammatory cell infiltration and pro-inflammatory cytokines production, improved cardiac functional recovery, alleviated cardiac injury, decreased apoptosis of cardiac cells and increased angiogenesis.

**Conclusion:**

In summary, we have demonstrated the therapeutic potential of IL-10 overexpressed BM-MSCs in the treatment of MI in diabetic mice.

**Electronic supplementary material:**

The online version of this article (10.1186/s13036-019-0163-6) contains supplementary material, which is available to authorized users.

## Background

Acute myocardial infarction (MI), commonly known as a heart attack, is a common cardiac emergency in which myocardial necrosis is caused by an unstable ischemic syndrome [1]. Currently the treatments to acute MI patients focuses on antiplatelet and antithrombotic treatment coupled with invasive assessment of coronary anatomy with a view to revascularization. Pharmacological therapies include β-blockade, statins and inhibitors of the renin-angiotensin-aldosterone axis [2]. Despite these therapeutic advances and promoted management of patients with acute myocardial infarction, MI remains a leading cause of death and disability worldwide as no treatment currently available is able to generate new contractile tissue or reverse ischemic myocardium [3]. Therefore, searching for more effective treatments to limit myocardial injury is still in urgent demand.

The prospect of developing novel treatments for acute MI that stimulate angiogenesis, promote myocardial regeneration and prevent left ventricular dysfunction has generated substantial interest in recent years. Stem cell therapy could promote cardiomyocyte regeneration and neovascularization, and recruit resident stem cells, which might ameliorate heart failure. Mesenchymal Stem Cells (MSCs) are non-hematopoietic population of bone marrow cells which are multipotent adult stem cells. BM-MSCs have been shown to be able to engraft and differentiate within the heart, which has revealed the potency to treat MI [4, 5].

Diabetes mellitus is a major risk factor for cardiovascular disease. It has been recognized that patients with diabetics suffered a greater mortality during the acute phase of MI and a higher morbidity in the post-infarction period [6]. The diabetic environment also affects the MSCs activities including inhibiting MSCs proliferation ability, suppressing their angiogenic and therapeutic potential for repairing [7, 8]. Although MSCs transplantation were widely evaluated to treat diabetes and MI, establishing more efficient and effective MSCs-related treatment are in urgent demand [9].

Interleukin-10 (IL-10) is an anti-inflammatory cytokine which has been shown to display protective function in cardiac dysfunction and inflammatory processes. Treatment of IL-10 in MI mice attenuated left ventricular (LV) dysfunction and decreased infarcted size in MI [10]. Our previous study also demonstrated that transplantation of mesenchymal stem cells overexpressing IL-10 attenuated cardiac impairments in MI rats [9]. IL-10 also suppressed the induction and progression of autoimmune pathogenesis associated with diabetes [11]. In current study, we overexpressed IL-10 in bone marrow derived MSCs (BM-MSCs) by using CRISPR/dCas9 activation system and evaluated the effects of transplantation of IL-10 overexpressed BM-MSCs on diabetic MI mice. Our studies may provide helpful information for guiding the development of stem-cell-based therapies in treating MI in diabetic patients.

## Material and methods

### Mice

Six weeks male C56BL/6 mice were obtained from SLAC (Shanghai, China) and housed 5 per cage. Mice were provided with distilled water and food ad libitum, and kept under a 12 h light/dark cycle at constant temperature (22.5 °C) and humidity (55%). The animal experiments were performed in accordance with the National Institutes of Health Guide for the Care and Use of Laboratory Animals and approved by Xijing Hospital, the Fourth Military Medical University.

### STZ model of diabetes

Streptozotocin (STZ) was purchased from Sigma (St. Louis, MO, USA). To establish Streptozotocin-induced diabetic mice, STZ was dissolved in a citrate buffer (pH 4.5), and injected intraperitoneally (40 mg/kg/d) for 5 consecutive days as described previously [12]. The control group received citrate buffer solution without STZ correspondingly. During STZ treatment, mice were provided with normal food and 10% sucrose water. On experimental day 6, switch the 10% sucrose water back to regular water. Three weeks post first STZ treatment, animals with random blood glucose value ≥300 mg/dL were defined as STZ-induced diabetic mice.

### Isolation and culture of mesenchymal stem cells from mouse bone marrow

Mesenchymal stem cells (MSCs)were isolated from bone marrow and cultured as described previously [13]. Briefly, bone marrow cells were harvested from tibia and femur of hind limb and suspended in DEME containing 10% fetal bovine serum (FBS) and Penicillin-Streptomycin (Thermo Fisher, Waltham, MA, USA). The cell suspension was filtered through a 70-μm cell strainer (Thermo Fisher) and then cultured at a density of 25 × 10^6^ cell/dish in 95 mm culture dish. After incubation at 37 °C in a humidified chamber with 5% CO_2_ for 3 h, the non-adherent cells were removed by changing the medium. After additional 8 h of culture, replaced the medium with fresh medium. Thereafter, replace the medium every 8 h for up to 72 h of initial culture. Then cells were cultured for 4–8 days and fresh medium was changed every 3 days. After 2 weeks of initiating culture, the cells were washed with phosphate buffered saline (PBS) and lifted by incubation with trypsin. All the lifted cells were cultured in 25-cm^2^ flask. Once cell confluence achieved, cells were harvested for further passage or for analysis.

### Heart cell isolation

Heart cells were isolated as described previously [14]. Briefly, 1000 U of heparin were injected into mice 10 min before cervical dislocation. The heart was rapidly removed, placed in a 37 °C water bath, and the aorta was cannulated with a 22-gauge needle connected to a modified Langendorff preparation. Then the heart was perfused and digested with 0.895 mg/ml collagenase type 2 and 0.5 mg/ml protease type XIV. The heart was placed into a Petri dish containing chilled staining buffer (2% FBS in PBS) and manually dispersed into a single cell suspension using razor blades. Single cell suspensions were sequentially filtered through 40-μm and 15-μm cell strainers.

### Flow cytometry

BM-MSCs were harvested and washed with PBS containing 2% FBS (Staining buffer) once. Cells were pre-blocked with anti-Fc receptor III/II monoclonal antibody (mAb) (clone 2.4G2) (Biolegend, San Diego, CA, USA) for 5 min and then cells were stained with FITC labeled anti-mouse CD29, CD44, CD90, CD34 and CD45 or corresponding isotype control antibody (Biolegend) on ice for 30 min. After wash with staining buffer for three times, the samples were analyzed by BD FACSCalibur flow cytometer and data was analyzed using FlowJo. In certain experiment, heart cells were pre-blocked with anti-Fc receptor III/II monoclonal antibody (mAb) and stained with APC labeled anti-mouse CD68 and FITC labeled anti-mouse CD11b (Biolegend).

### Overexpression of IL-10 in BM-MSCs from diabetic mice

The dCas-Synergistic activation mediator (dCas-SAM) system was used to overexpress IL-10 in BM-MSCs. The gRNA sequence for IL10 or control was designed using online CRISPR design tool from CRISPR.mit.edu platform (http://tools.genome-engineering.org). Total 6 gRNA sequences with high scores were cloned into dCas9-VP64-MS2 plasmid as described previously [15]. Then the 1.0 nM pCas9/gIL10 or pCas9/gCtrl was co-transfected with 1.0 nM plasmid MS2-p65-HSF1 which encodes MS2-P65-HSF1 transcriptional activation complex into diabetic BM-MSCs using Lipofectamine 2000 following manufacture’s protocol. 48 h post transfection, cells were harvested and analyzed for IL-10 production. The gRNA with best efficiency to activate IL-10 expression was used for future study.

### Elisa

BM-MSCs were seeded in 12-well plate with 10 × 10^6^ cell/well. 48 h later, cell culture supernatant was collected and the IL-10 level was determined using commercial ELISA kit (R&D systems, Minneapolis, MN, USA) following manufactures’ protocol. In certain experiments, the peri-infarct area in the hearts was isolated and homogenized in cell extraction buffer (Thermo Fisher) following manufactures’ protocol. The IL-10 level in homogenates was measured using ELISA kit.

### qRT-PCR

The total RNA from BM-MSCs or heart tissue was isolated using an RNeasy Mini kit (Qiagen, CA, USA) according to the manufacturer’s instructions. Reverse transcription was performed using SuperScript® III First-Strand Synthesis System (Thermo Fisher, Waltham, MA, USA). Real time quantitative PCR reactions were set up in triplicate with SYBR® Green Master Mix (Bio-Rad, Hercules, CA, USA) and run on a QuantStudio 3 Real-Time PCR System (Thermo Fisher, USA). The following primers were used in the current study: IL-10 Forward: 5′- GCCTTATCGGAA ATGATCCA -3′, Reverse: 5′- TTTTCACAGGGGAGAAATCG -3′. GAPDH Forward: 5′- AACTTTGGCATTGTGGAAGG-3′, Reverse: 5′- ACACATTGGGGGTAGGAACA-3′. IL-1β Forward: 5′- AACCTGCTGGTGTGTGACGTTC -3′, Reverse: 5′- CAGCACGAGGCT TTTTTGTTGT -3′. IL-6 Forward: 5′- ACAACCACGGCCTTCCCTACTT -3′, Reverse: 5′-CACGATTTCCCAGAGAACATGTG -3′. MCP-1 Forward: 5′ -CCACTCACCTGCTGCTA CTCAT -3′, Reverse: 5′ –TGGTGATCCTCTTGTAGCTCTCC -3′. TNF-α Forward: 5′ -GCCTCTTCTCATTCCTGCTTG -3′, Reverse: 5′ -CTGATGAGAGGGAGGCCATT -3′.

### Western blot

Total proteins of MSCs or peri-infarct area tissue were extracted using Total Protein Extraction Kit (Novus Biologicals, LLC, Centennial, CO, USA). The protein concentration was measured using Bio-Rad Protein Assay (Bio-Rad, USA). Total 20 μg proteins were loaded onto SDS-PAGE gel and transferred to PVDF membrane. The membrane was blocked with 5% non-fat milk at room temperature for 1 h and then incubated with primary antibodies overnight. Next day, membranes were washed with wash buffer (Thermo Fisher) for 3 times and then incubated with corresponding HRP-conjugated secondary antibodies at room temperature for 1 h. Primary antibodies used in current study were: anti- IL10 (Abcam, Cambridge, MA, USA), anti- β actin (Sigma, St Louis, MO, USA).

### Myocardial infarction (MI) mice and treatment

MI was induced in mice by surgical occlusion of the left anterior descending artery through a left anterolateral approach as described previously [16]. All surgeries were performed under sodium pentobarbital anesthesia to minimize pains, and in compliance with the protocol approved by the Committee on the Ethics of Animal Experiments of Xijing Hospital, Fourth Military Medical University. In brief, ligation of the left anterior descending artery (LAD) was performed in anesthetized animals after the chest was opened. The position was 1 to 2 mm distal to the line between the left border of the pulmonary conus and the right border of left atrial appendage [9]. The cells were transplanted 1 h after the ligation by intramyocardial injection. Seven days post transplantation, mice were evaluated.

### Dissection of the heart and histological analysis of infarct area

Hearts were obtained hearts were fixed in 10% formalin and mounted in paraffin. For immunohistochemistry, blocks were cut into 5 μm thick sections, and mounted on glass slides for staining. Slides were deparaffinized, and subjected in hot citric acid buffer for antigen retrieval. After cooling, slides were permeabilized with 0.2% Triton-100 for 15 min and were blocked with 1% BSA in PBS for 2 h. Slides were incubated with primary antibodies (anti-CD68 or anti-vWF) at 4 °C for overnight. Primary antibodies were visualized with corresponding secondary antibodies conjugated with Alexa Fluor 488 or Alexa Fluor 647. Nuclei were stained with DAPI (Thermo Fisher). Micrographs of all immunostains were acquired via Olympus BX51 Fluorescence Microscope with camera. To monitor the apoptosis, the In-Situ Cell Death Detection Kit, Fluorescein (Thermo Fisher) was used following manufacture’s protocol. Fibrosis was evaluated by Masson’s trichrome staining as described previously [17].

### Statistical analysis

Two-way ANOVA analysis and student *t*-test were used. Statistical difference was considered as significant only if *p* < 0.05.

## Results

### Overexpression of IL10 in bone marrow-derived mesenchymal stem cells (BM-MSCs)

**Fig. 1:**
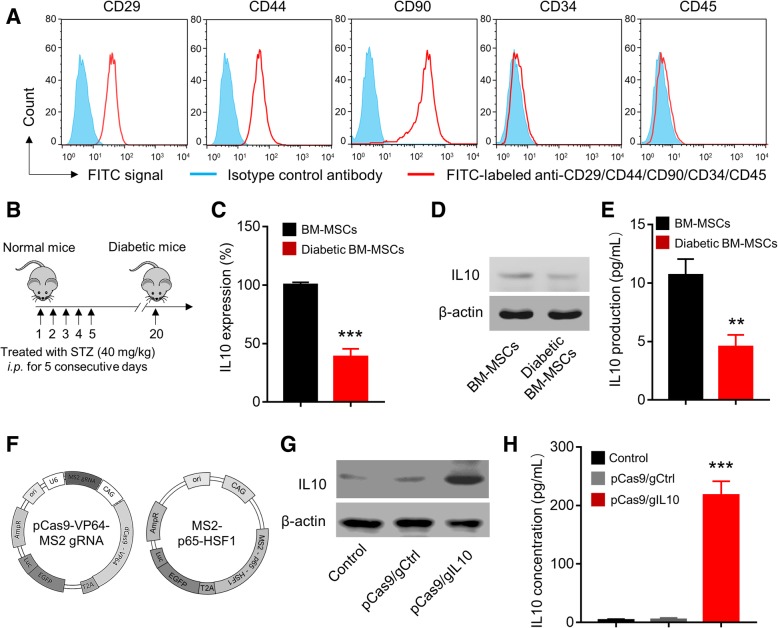
CRISPR/dCas9 activation (CRISPRa) system mediated overexpression of IL10 in Bone Marrow-Derived Mesenchymal Stem Cells (BM-MSCs). **a** Immunophenotypic profile of BM-MSCs. Flow cytometry histograms after three passages show the expression of selected surface molecules (including CD29, CD44, CD90, CD34 and CD45). Cells were stained with FITC-labeled antibodies or isotype control antibody. The BM-MSCs were positive for CD29, CD44, and CD90 but negative for CD34 and CD45. **b** Establishment of streptozotocin-induced diabetic model. 6–8-week-old BALB/c mice were intraperitoneal (*i.p.*) injection with Streptozotocin (STZ) (40 mg/kg) for 5 consecutive days. The IL-10 expressions in mRNA level (**c**) and protein level (**d**) of BM-MSCs isolated from healthy mice and streptozotocin (STZ)-induced diabetic mice was examined by real-time PCR (RT-PCR) and Western blotting, respectively. Data represent means ± SD. ***p < 0.001, *n* = 3. **e** Production of IL-10 by BM-MSCs and diabetic BM-MSCs. The IL-10 levels in the supernatant were measured by enzyme-linked immunosorbent assay (ELISA). Data represent means ± SD. **p < 0.005, *n* = 3. **f** Schematic diagram of CRISPR/dCas9 activation plasmids. The engineered plasmid dCas9-VP64-MS2 gRNA encodes Cas9 that lacked nuclease activity (dCas9) and gRNA, the plasmid MS2-p65-HSF1 encodes MS2-P65-HSF1 transcriptional activation complex. The gRNA Sequence was designed by the online CRISPR Design Tool (http://tools.genome-engineering.org). BM-MSCs isolated from diabetic mice were transfected with CRISPRa plasmids expressing IL10 (pCas9/gIL10) or control plasmids (pCas9/gCtrl). The IL10 expression (**g**) and IL10 production (**h**) in diabetic BM-MSCs were examined by Western blotting and ELISA 48 h post transfection. Data represent means ± SD. ***p < 0.001, *n* = 3

To establish the cell model of BM-MSCs overexpressing IL10, first we isolated and cultured the mesenchymal stem cells from mouse bone marrow and monitored the expression of cell surface markers. As shown in Fig. 1a, BM-MSCs were CD29, CD44, and CD90 positive while were CD34 and CD45 negative. These results demonstrated that the BM-MSCs we made had the typical markers of MSCs, indicating the successful differentiation of bone marrow cells to MSCs [18]. IL-10 has been shown to play a protective role in diabetes and decreased IL-10 level was associated with diabetes [19]. We continued to detect the IL-10 expression in BM-MSCs from wild type and diabetic mice. The diabetic mice were induced by injection of Streptozotocin as described in Fig. 1b and methods. As shown in Fig. 1c, we identified significantly decreased IL-10 mRNA in diabetic BM-MSCs when compared to normal BM-MSCs. Consistently, we detected decreased IL-10 protein level in diabetic BM-MSCs (Fig. 1d) and significantly decreased IL-10 in cell culture supernatant of diabetic BM-MSCs (Fig. 1e) when compared to those of normal BM-MSCs. As IL-10 played important protective role in diabetes, we utilized the CRISRP/dCas9 activation system to overexpress IL-10 in BM-MSCs (Fig. 1f). The gRNAs (gIL10) were designed using the CRISPR.mit.edu platform and 6 gRNAs with high scores were cloned into dCas9-VP64-MS2 plasmid (Additional file 1: Figure S1A). After transfection of CRISPRa plasmids expressing IL10 gRNA together with MS2-p65-HSF1 (we termed pCas9/gIL10) into BM-MSC, the gRNA with best efficiency to activate IL-10 mRNA expression was used for future study (Additional file 1: Figure S1B). Correspondingly, we detected significantly increased IL-10 protein in diabetic BM-MSCs (Fig. 1g) and in cell culture supernatant (Fig. 1h). In contrast, transfection of control plasmid which expressed control gRNA (pCas9/gCtrl) did not affect IL-10 expression. Therefore, we successfully established the cell model of diabetic BM-MSCs which can overexpress IL-10.

### Transplantation of IL-10 overexpressed diabetic BM-MSCs induces increased expression of IL10 in diabetic myocardial infarction mice

**Fig. 2:**
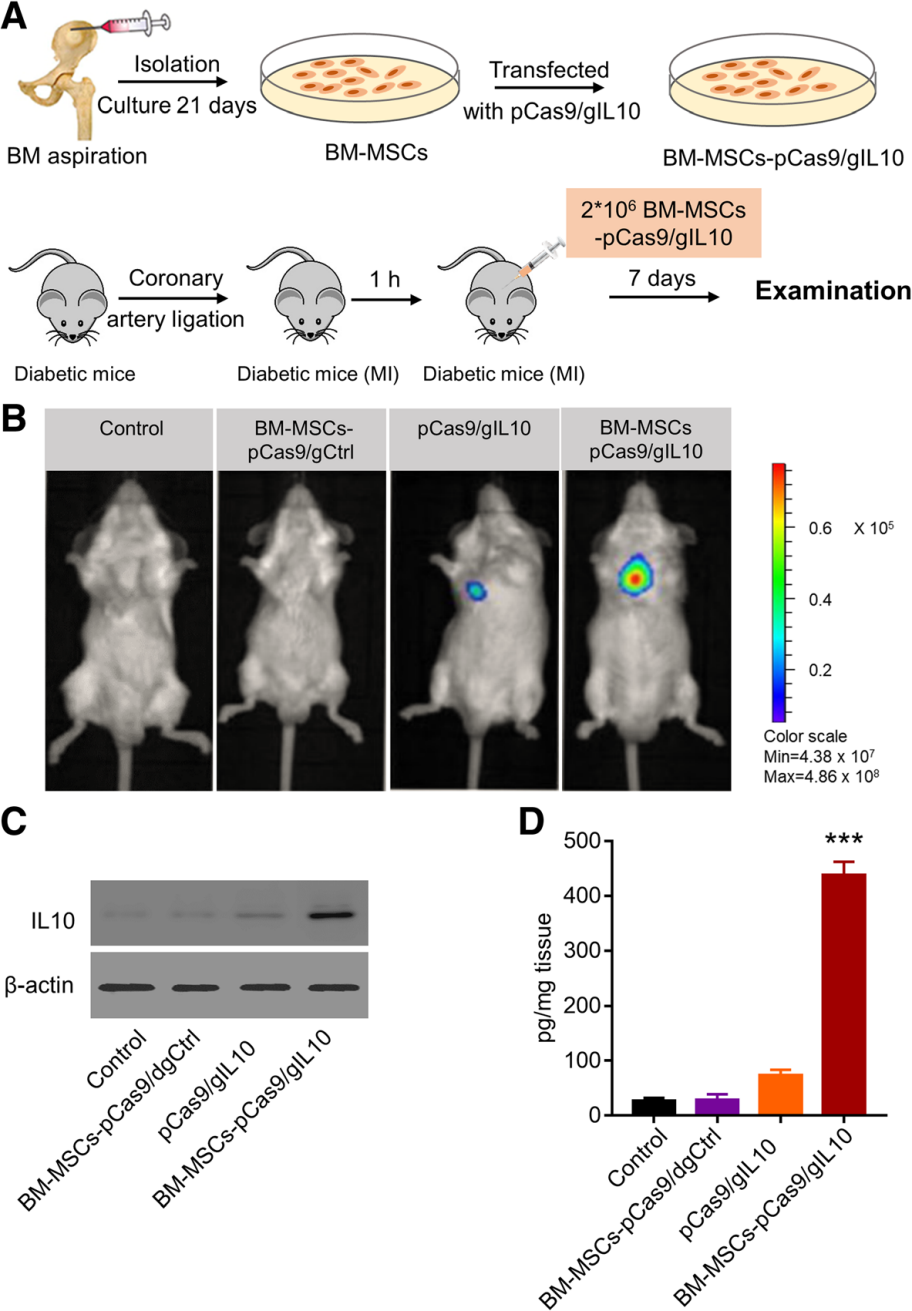
BM-MSCs-pCas9/gIL10 transplantation induces increased expression of IL-10 in diabetic mice with myocardial infarction. **a** Schematic depicts the experiment protocol. BM-MSCs were isolated and cultured, and then transfected with pCas9/dgCtrl or pCas9/dgIL10. Mice models of diabetic MI were established via coronary artery ligation. STZ-induced diabetic mice were injected with BM-MSCs-pCas9/dgCtrl, pCas9/dgIL10 or BM-MSCs pCas9/dgIL10 at 2 sites near the border zone of infarction (medial and lateral zones) 1 h post myocardial infarction (MI) accomplishment, and 7 days later the mice were subjected to examination. **b** In vivo optical bioluminescence imaging (BLI). Luciferase gene was inserted into plasmids, and BLI was detected using the Xenogen In Vivo Imaging System 1-weeks post transplantation by intravenously injecting luciferase substrate. Four groups were treated as follows: Control (PBS only), BM-MSCs-pCas9/gCtrl (BM-MSCS transfected with control CRISPR/Cas9 plasmids), pCas9/gIL10 (direct injection of CRISPR/Cas9 plasmids express IL-10), and BM-MSCs-pCas9/gIL10 (BM-MSCS transfected with control CRISPR/Cas9 plasmids express IL10). The number of cells was 2*10^6^, and the mass of pCas9/dgIL10 was 40 pmol. **c** The IL10 expressions in peri-infarct area in the control MI heart or hearts treated with BM-MSCs pCas9/gCtrl, pCas9/gIL10 or BM-MSCs pCas9/gIL10 were detected using Western blotting 1-week post transplantation. β-actin was used as a loading control. **d** The production of IL-10 in in peri-infarct area was examined by ELIAS 1-week post transplantation. Data represent means ± SD. ***p < 0.001, *n* = 8

We next transplanted the IL-10 overexpressed diabetic BM-MSCs to diabetic myocardial infarction (MI) mice as presented in Fig. 2a. Then we monitored the IL-10 expression in vivo. The CRISPR Activation system plasmids also encoded the luciferase (Fig. 1e), therefore we monitored the luciferase activity by intravenously injecting luciferase substrate 1-week post transplantation and then analyzed by in vivo optical bioluminescence imaging. As shown in Fig. 2b, in both control mice and mice transplanted with BM-MSCs transfected with pCas9/gCtrl, there was no obvious luciferase activity detected. In contrast, in mice directly injected with pCas9/gIL10 plasmids, there was minimal luciferase activity detected, indicating that some cells may uptake the plasmids and enable IL-10 expression. However, the efficiency was very limited. Interestingly, we detected robust and obvious luciferase activity in mice transplanted with BM-MSCs which were transfected with pCas9/gIL10 plasmids (we termed BM-MSCs-pCas9/gIL10). Correspondingly, we detected obvious increased IL-10 protein in peri-infart area tissue of mice transplanted with BM-MSCs-pCas9/gIL10 by western blot (Fig. 2c), and significantly increased IL-10 in tissue homogenates by ELISA (Fig. 2d) when compared to control mice. Taken together, our data demonstrated that transplantation of BM-MSCs expressing IL-10 enabled in vivo expression of IL-10 in diabetic myocardial infarction mice.

### Transplantation of BM-MSCs-pCas9/gIL10 suppresses inflammatory cells infiltration and pro-inflammatory cytokines expression in the myocardium

**Fig. 3:**
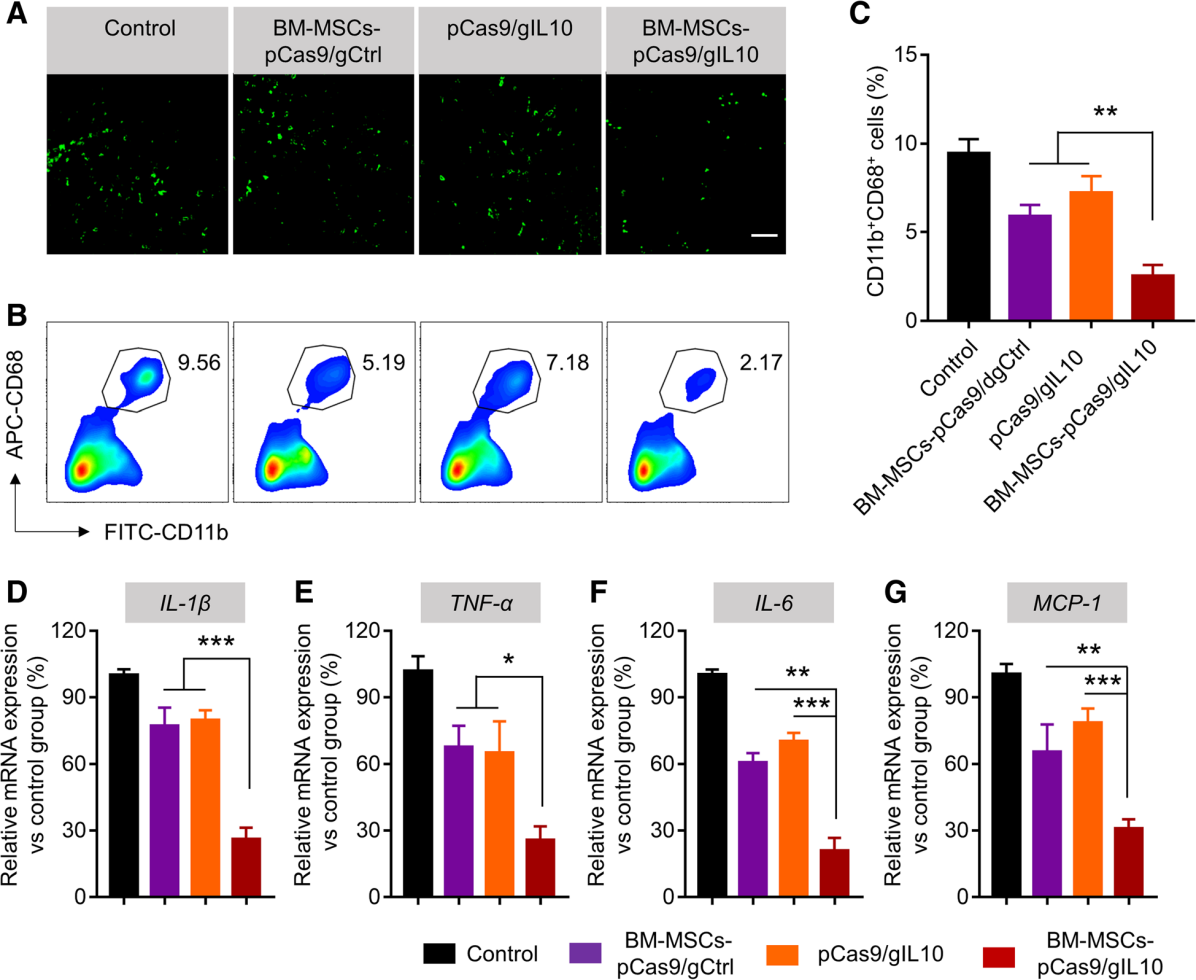
Transplantation of BM-MSCs-pCas9/gIL10 suppresses infiltration of inflammatory cells and expression of proinflammatory cytokines in the myocardium. **a** Representative fluorescent micrographs show the presence of inflammatory cells (CD68-positive, green fluorescence) peri-infarct area in the control MI heart or hearts treated with BM-MSCs pCas9/gCtrl, pCas9/gIL10 or BM-MSCs pCas9/gIL10. The examination was performed 1-week post transplantation. Scale bar was 20 μm. **b** Flow cytometry analysis of infiltration of CD11b^+^CD68^+^ cells peri-infarct area in the heart. Tissues from control MI heart or hearts treated with BM-MSCs-pCas9/gCtrl, pCas9/gIL10 or BM-MSCs-pCas9/gIL10 were digested into single cells stained with FITC labeled CD11b and APC labeled CD68 antibodies, and then examined by flow cytometry. **c** Bar graph shows quantitative analysis of infiltrating CD68-positive cells. Data represent means ± SD. **p < 0.005, *n* = 8. **d**-**g** Quantitative analysis of mRNA expression of proinflammatory cytokines and chemokines (IL-1β, TNF-α, IL-6 and MCP-1) in the border zone of LV infarct of LV infarct at 1-week post-MI. mRNA expression normalized to GAPDH expression. Data represent means ± SD. *p < 0.05, **p < 0.005, ***p < 0.001, *n* = 8

Inflammation and inflammatory cell infiltration are the hallmarks of MI [20]. As IL-10 is well-known for its anti-inflammatory activities [21], we continued to detect the effect of IL-10 expression on inflammation in the heart. First the immunohistochemical staining of CD68-positive cells on cardiac tissue sections was carried out to study the inflammatory cell infiltration post MI. We detected obvious CD68^+^ cells (macrophages and monocytes) in the border zone of left ventricular (LV) infarct after MI (Fig. 3a) in normal/non-treated mice. Similar level of CD68^+^ cells were detected in mice transplanted with BM-MSCs-pCas9/gCtrl or directly injected with pCas9/gIL-10 plasmids, indicating transplantation of BM-MSCs-pCas9/gCtrl or injection of pCas9/gIL-10 did not affect inflammatory cells infiltration. In contrast, in MI mice transplanted with BM-MSCs-pCas9/gIL-10, we detected less CD68^+^ cells, indicating transplantation of BM-MSCs-pCas9/gIL-10 suppressed inflammatory cells infiltration. As BM-MSCs-pCas9/gIL-10 overexpressed IL-10, these results suggested that IL-10 suppressed inflammatory cells infiltration after MI. Similarly, significantly decreased frequency of CD68^+^ CD11b^+^ cells was detected in peri-infarct area of mice transplanted with BM-MSCs-pCas9/gIL-10 when compared to mice transplanted with BM-MSCs-pCas9/gCtrl or directly injected with pCas9/gIL-10 plasmids (Fig. 3b & c). These results demonstrated that Transplantation of BM-MSCs-pCas9/gIL10 suppressed inflammatory cells infiltration. We next evaluated the effects of transplantation on pro-inflammatory cytokines production in peri-infarct area after transplantation. We monitored the mRNA levels of IL-1β, TNF-α, IL-6 and MCP-1 in border zone of left ventricular infarct by RT-PCR. We detected significantly decreased the mRNA levels of IL-1β (Fig. 3d), TNF-α (Fig. 3e), IL-6 (Fig. 3f) and MCP-1 (Fig. 3g) in border zone of left ventricular infarct in mice transplanted with BM-MSCs-pCas9/gIL-10 when compared to border zone in mice transplanted with BM-MSCs-pCas9/gCtrl or directly injected with pCas9/gIL-10 plasmids. Taken together, our data demonstrated that Transplantation of BM-MSCs-pCas9/gIL10 suppresses inflammatory cells infiltration and pro-inflammatory cytokines expression in the myocardium.

### Transplantation of BM-MSCs-pCas9/gIL10 improves cardiac functional recovery in diabetic myocardial infarction mice

**Fig. 4:**
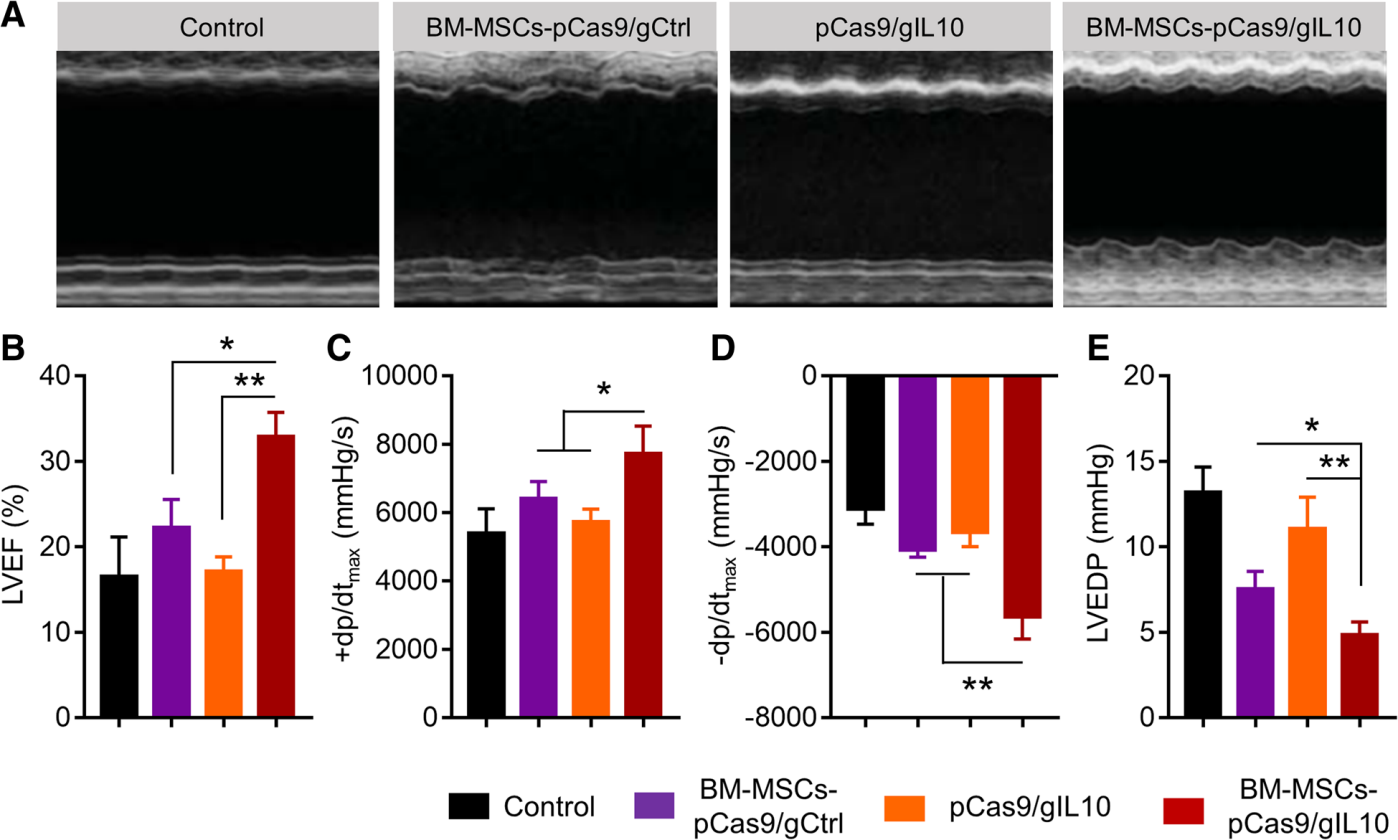
Transplantation of BM-MSCs pCas9/gIL10 improves cardiac functional recovery in mice model of diabetic MI. **a** Representative echocardiographic images 4 weeks after MI and treated with BM-MSCs-pCas9/gCtrl, pCas9/gIL10 or BM-MSCs-pCas9/gIL10. **b** Left ventricular ejection fraction (LVEF). Data represent means ± SD. *p < 0.05, **p < 0.005, *n* = 8. **c**-**e** Hemodynamic analyses including +dp/dtmax, −dp/dtmax, and LV end diastolic pressure (LVEDP). Data represent means ± SD. *p < 0.05, **p < 0.005, *n* = 8

First, the situation of engraftment was examined. GFP positive cells were observed in the BM-MSCs-pCas9/gIL10 group (Additional file 1: Figure S2), indicating that at least partial BM-MSCs were engrafted and could be alive for 4 weeks post transplantation. Inflammatory response has been implicated in the pathogenesis of post-infarction remodeling and heart failure [22]. As transplantation of BM-MSCs-pCas9/gIL-10 suppressed inflammatory response, we continued to evaluate its effects on cardiac function by using echocardiography and left ventricular (LV) catheterization. As shown in Fig. 4a & b, transplantation of BM-MSCs-pCas9/gIL-10 increased LV ejection fraction significantly more than transplantation of BM-MSCs-pCas9/gCtrl or directly injection of pCas9/gIL-10 plasmids. Transplantation of BM-MSCs-pCas9/gIL-10 also increased maximal values (+dP/dtmax) and minimum values (−dP/dtmin) of the instantaneous first derivative of LV pressure, and LV end-diastolic pressure (LVEDP) significantly more than transplantation of BM-MSCs-pCas9/gCtrl or directly injection of pCas9/gIL-10 plasmids (Fig. 4c-e). Therefore, our data demonstrated that transplantation of BM-MSCs-pCas9/gIL10 improves cardiac functional recovery in diabetic MI mice.

### Transplantation of BM-MSCs-pCas9/gIL10 alleviates cardiac injury, decreases apoptosis of cardiac cells and increases angiogenesis in diabetic MI mice

**Fig. 5:**
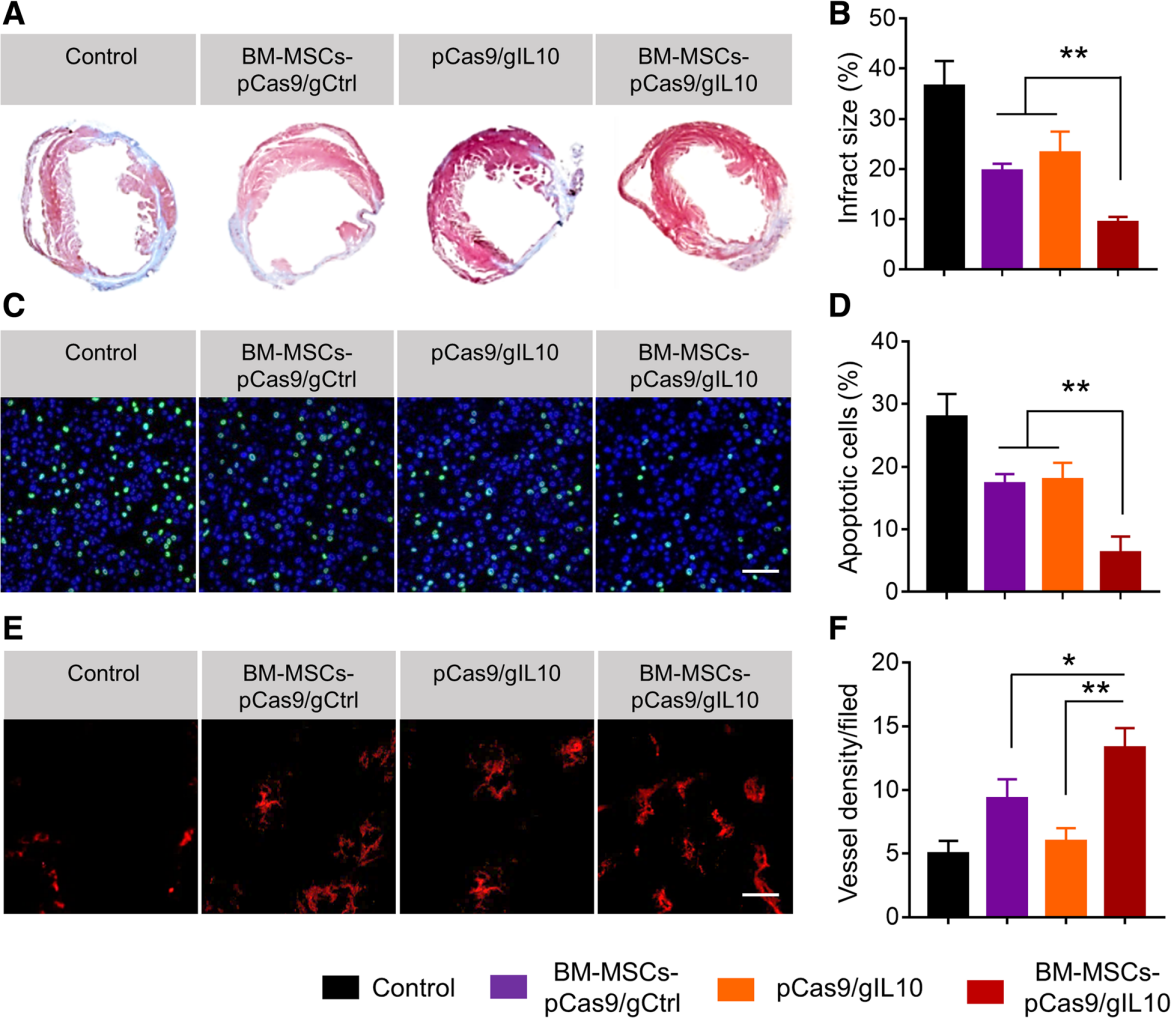
Transplantation of BM-MSCs-pCas9/gIL10 alleviates cardiac injury, decreases apoptosis of cardiac cells and increases angiogenesis in mice model of diabetic myocardial infarction. **a** Representative Masson’s trichrome–stained myocardial sections in the control MI heart or hearts treated with BM-MSCs pCas9/gCtrl, pCas9/gIL10 or BM-MSCs pCas9/gIL10. Blue, scar tissue; red, viable myocardium. **b** Quantitation of infarct size in the four indicated experimental groups. Data represent means ± SD. **p < 0.005, *n* = 8. **c** Representative fluorescent micrographs showing the presence of TUNEL+ (terminal deoxynucleotidyl transferase–mediated deoxyuridine triphosphate nick end labeling–positive) apoptotic cells (green) in the border zone of LV infarct. Nuclei were stained by DAPI (blue). **d** Quantification of percentage of apoptotic cells in the four indicated experimental groups. Data represent means ± SD. **p < 0.005, *n* = 8. **e** Angiogenesis was inspected using von Willebrand factor (vWF) staining in heart sections from different groups 4 weeks after MI and transplantation. Tissue sections were stained primary rabbit anti-vWF antibody and secondary goat anti-Rabbit IgG antibody labeled with Alexa Fluor 647 (red). **f** Summary of total vessel density in different groups. Data represent means ± SD. *p < 0.05, **p < 0.005, *n* = 8

As transplantation of BM-MSCs-pCas9/gIL10 improved the cardiac function, we continued to explore the underlying mechanisms. First Masson’s trichorome staining was performed to analyze the infarct injury. In Fig. 5a, there were typical representations of Masson’s trichrome staining of hearts (blue color staining) from mice with different treatments. More relative staining pictures were shown in Additional file 1: Figure S3. We found that the scar tissue area of mice transplanted with BM-MSCs-pCas9/gIL10 was obvious decreased when compared to hearts from mice transplanted with BM-MSCs-pCas9/gCtrl or directly injected with pCas9/gIL-10 plasmids. After quantitation of scar area by ImageJ, we found the difference was significant (Fig. 5b). Therefore, transplantation of BM-MSCs-pCas9/gIL10 significantly alleviated cardiac injury in diabetic MI mice. It has been described that IL-10 prevented apoptosis in multiple diseases including MI [10, 23, 24]. Thus, we continued to evaluate the effect of transplantation of BM-MSCs-pCas9/gIL10 on cardiac cells apoptosis. As shown in Fig. 5c, obvious apoptotic cells were detected in border zone of LV infarct in diabetic MI mice. In diabetic MI mice transplanted of BM-MSCs-pCas9/gIL10, the apoptotic cells were significantly less than that in MI mice transplanted with BM-MSCs-pCas9/gCtrl or directly injected with pCas9/gIL-10 plasmids (Fig. 5c & d). Angiogenesis plays important role in heart function recovery in MI [25]. Therefore, we tested whether transplantation of BM-MSCs-pCas9/gCtrl also affect angiogenesis in diabetic MI mice. Angiogenesis was evaluated by staining von Willebrand factor (vWF), a commonly used indicator of angiogenesis [26]. As shown in Fig. 5e, we detected more staining of vWF in heart sections from mice transplanted with BM-MSCs-pCas9/gIL-10 when compared to that from mice transplanted with BM-MSCs-pCas9/gCtrl or directly injected with pCas9/gIL-10 plasmids. After quantitation, we found the difference was significant (Fig. 5f). Collectively our data demonstrated that transplantation of BM-MSCs-pCas9/gIL10 alleviated cardiac injury, decreased apoptosis of cardiac cells and increased angiogenesis in diabetic MI mice.

## Discussion

Myocardial infarction (MI) is a common cause of mortality in people with diabetes. Stem cell-mediated therapies have been widely evaluated and utilized for the treatment of MI. Mesenchymal stem cells (MSCs) have been reported to repair damaged myocardium and improve cardiac function after myocardial infarction (MI) in pre-clinical studies [27, 28]. However, due to the persistent inflammatory environment in infarcted myocardium and diabetes, the survival, differentiation of transplanted MSCs and their therapeutic benefit needs to be improved. Therefore, novel approaches for designing MSC protection under the hostile microenvironment of injured myocardium are necessary for successful application. IL-10 is an anti-inflammatory cytokine which has been proven to have beneficial effects on MI. However, low levels of IL-10 have been considered as a risk factor of diabetes, which may also contribute to MI in diabetic patients. In current study, we utilized CRISPR/dCas9 activation system to establish IL-10 overexpressed BM-MSCs and tested the role of these BM-MSCs in diabetic MI mice. We reported that transplantation of BM-MSCs expressing IL-10 increased the IL-10 expression in vivo, suppressed inflammatory response in myocardium, and improved cardiac function.

MI is the leading cause of death around the world which results in adverse LV remolding and cardiac dysfunction. MI triggered inflammatory reactions accompanied by cytokine release and inflammatory cells infiltration into the myocardial area, which contributed to tissue necrosis and organ dysfunction during MI. IL-10 has been shown to suppress infiltration of inflammatory cells and expression of inflammatory cytokines in the myocardium [10].The protective roles of IL-10 in MI suggested that IL-10 could be a potential treatment for MI. Previously our group also demonstrated that MSCs overexpressing IL-10 attenuated cardiac impairments in MI rats, confirming the potentials of IL-10 plus stem cell therapy to treat MI. However, the diabetic conditions affected the MSCs activities and we detected significantly decreased IL-10 production in BM-MSCs from diabetic mice. Therefore, we compensated the IL-10 expression in diabetic BM-MSCs using the dCas9 activation system (Fig. 1).

CRISPR activation (CRISPRa) is one type of CRISPR tool that use modified versions of dCas9, a mutation of Cas9 without endonuclease activity, with added transcriptional activators on dCas9 or the guide RNAs (gRNAs). By utilizing MS2, p65, and HSF1 proteins, dCas9-SAM system recruits various transcriptional factors working synergistically to activate the gene of interest [29]. In current study, we demonstrated that using CRISPRa, we successfully overexpressed IL-10 in diabetic BM-MSCs. The expression of IL-10 by CRISPRs was very stable as we also detected obvious expression of IL-10 in mice after transplantation of BM-MSCs expressing IL-10 (Fig. 2). Besides CRISPRa, several other strategies have been utilized to overexpress IL-10 in BM-MSCs including AAV overexpression [30], retroviral transduction [31]. The advantage of using CRISPRa is that CRISPR will allow to study genes in their native context and get more biologically relevant results.

Transplantation of IL-10 expressing MSCs has been shown to attenuate cardiac impairments in MI rats [9]. In current study, we also demonstrated that CRISPRa engineered BM-MSCs which overexpressed IL-10 protected against myocardial infarction in diabetic mice too. Our current and previous study strongly suggested MSCs-based stem cell therapy could be a very potential treatment for MI. Ischemia/reperfusion of myocardium results in a significant enhancement of inflammatory response including inflammatory cells infiltration and production of inflammatory cytokines including IL-1β, TNF-α, IL-6 and MCP-1, which contributes to tissue necrosis and mediates organ dysfunction. Transplantation of IL-10 overexpressing BM-MSCs significantly blocked CD68^+^ inflammatory cells infiltration and productions of IL-1β, TNF-α, IL-6 and MCP-1. These inhibitory effects majorly depended on the IL-10 expression in these BM-MSCs as transplantation of BM-MSCs which did not overexpress IL-10 did failed to inhibit neither inflammatory cells infiltration nor inflammatory cytokines production (Fig. 3). In addition, transplantation of IL-10 overexpressing BM-MSCs significantly prevented cell apoptosis and enhanced angiogenesis. These effects also depended on IL-10 expression in BM-MSCs (Figs. 4 & 5)). All our findings were consistent with previous descriptions of IL-10 actives, which included inhibiting apoptosis of multiple cell types [9, 32, 33] and inducing angiogenesis [34, 35].

The mechanisms underlying the effects of IL10 overexpression in MSC therapy for MI animals need to be further characterized. For example, oxidative stress has been implicated in MI. As IL-10 has been shown to protect against oxidative stress [36], the anti-oxidative stress activity of IL-10 expression in BM-MSCs should also contribute to the protective activity against MI.

In summary, we have demonstrated the therapeutic potential of IL-10 overexpressed BM-MSCs in the treatment of MI in diabetic mice. The infarct size and cardiac function recovery can be significantly promoted by transplantation of IL-10 overexpressed BM-MSCs. Sustained cell survival, attenuated inflammation and enhanced angiogenesis contributed to the functional improvement of cardiac regeneration after MI.

We established IL10-overexpressing bone marrow-derived mesenchymal stem cells by CRISPRa system engineering. Transplantation of these BM-MSCs could be used for the treatment of myocardial infarction in diabetic mice.

## Additional file

Additional file 1: **Figure S1.** (A) gRNAs (gIL10) were designed using the CRISPR.mit.edu platform, and 6 candidate gRNA sequence were selected according to the scores. (B) Activation of IL10 expression after transfected with different pCas9/gIL10. The activation of IL10 were examined by real-time PCR. The Guide#2 was selected for the further experiments. **Figure S2.** Representative confocal laser microscopic images of engrafted BM-MSCs at 4 weeks after transplantation. GFP positive cells indicated the alive BM-MSCs. **Figure S3.** The five sections of the representative Masson’s trichrome–stained myocardial sections in the control MI heart or hearts treated with BM-MSCs pCas9/gCtrl, pCas9/gIL10 or BM-MSCs pCas9/gIL10. Blue, scar tissue; red, viable myocardium. (PDF 233 kb)

## Abbreviations

IL-10: Interleukin-10
MI: Myocardial infarction
MSC: Mesenchymal stem cell

## References

[1] Anderson JL, Morrow DA (2017). Acute myocardial infarction. N Engl J Med.

[2] Steg PG, James SK, Atar D, Badano LP, Blomstrom-Lundqvist C, Borger MA, Di Mario C, Dickstein K, Ducrocq G, Task Force on the management of STseamiotESoC (2012). ESC guidelines for the management of acute myocardial infarction in patients presenting with ST-segment elevation. Eur Heart J.

[3] Spath NB, Mills NL, Cruden NL (2016). Novel cardioprotective and regenerative therapies in acute myocardial infarction: a review of recent and ongoing clinical trials. Futur Cardiol.

[4] Williams AR, Hatzistergos KE, Addicott B, McCall F, Carvalho D, Suncion V, Morales AR, Da Silva J, Sussman MA, Heldman AW, Hare JM (2013). Enhanced effect of combining human cardiac stem cells and bone marrow mesenchymal stem cells to reduce infarct size and to restore cardiac function after myocardial infarction. Circulation.

[5] Wen Z, Zheng S, Zhou C, Wang J, Wang T (2011). Repair mechanisms of bone marrow mesenchymal stem cells in myocardial infarction. J Cell Mol Med.

[6] Jacoby RM, Nesto RW (1992). Acute myocardial infarction in the diabetic patient: pathophysiology, clinical course and prognosis. J Am Coll Cardiol.

[7] Kim H, Han JW, Lee JY, Choi YJ, Sohn YD, Song M, Yoon YS (2015). Diabetic mesenchymal stem cells are ineffective for improving limb ischemia due to their impaired Angiogenic capability. Cell Transplant.

[8] De Rosa S, Arcidiacono B, Chiefari E, Brunetti A, Indolfi C, Foti DP (2018). Type 2 diabetes mellitus and cardiovascular disease: genetic and epigenetic links. Front Endocrinol (Lausanne).

[9] Meng X, Li J, Yu M, Yang J, Zheng M, Zhang J, Sun C, Liang H, Liu L (2018). Transplantation of mesenchymal stem cells overexpressing IL10 attenuates cardiac impairments in rats with myocardial infarction. J Cell Physiol.

[10] Krishnamurthy P, Rajasingh J, Lambers E, Qin G, Losordo DW, Kishore R (2009). IL-10 inhibits inflammation and attenuates left ventricular remodeling after myocardial infarction via activation of STAT3 and suppression of HuR. Circ Res.

[11] Pennline KJ, Roque-Gaffney E, Monahan M (1994). Recombinant human IL-10 prevents the onset of diabetes in the nonobese diabetic mouse. Clin Immunol Immunopathol.

[12] Wu KK, Huan Y (2008). Streptozotocin-induced diabetic models in mice and rats. Curr Protoc Pharmacol.

[13] Soleimani M, Nadri S (2009). A protocol for isolation and culture of mesenchymal stem cells from mouse bone marrow. Nat Protoc.

[14] Afanasyeva M, Georgakopoulos D, Belardi DF, Ramsundar AC, Barin JG, Kass DA, Rose NR (2004). Quantitative analysis of myocardial inflammation by flow cytometry in murine autoimmune myocarditis: correlation with cardiac function. Am J Pathol.

[15] Liao HK, Hatanaka F, Araoka T, Reddy P, Wu MZ, Sui Y, Yamauchi T, Sakurai M, O'Keefe DD, Nunez-Delicado E (2017). In vivo target gene activation via CRISPR/Cas9-mediated trans-epigenetic modulation. Cell.

[16] Gao E, Koch WJ (2013). A novel and efficient model of coronary artery ligation in the mouse. Methods Mol Biol.

[17] Tae HJ, Park SM, Cho JH, Kim IH, Ahn JH, Park JH, Won MH, Chen BH, Shin BN, Shin MC (2016). Differential activation of cFos in the paraventricular nuclei of the hypothalamus and thalamus following myocardial infarction in rats. Mol Med Rep.

[18] Secunda R, Vennila R, Mohanashankar AM, Rajasundari M, Jeswanth S, Surendran R (2015). Isolation, expansion and characterisation of mesenchymal stem cells from human bone marrow, adipose tissue, umbilical cord blood and matrix: a comparative study. Cytotechnology.

[19] Barry JC, Shakibakho S, Durrer C, Simtchouk S, Jawanda KK, Cheung ST, Mui AL, Little JP (2016). Hyporesponsiveness to the anti-inflammatory action of interleukin-10 in type 2 diabetes. Sci Rep.

[20] Liu J, Wang H, Li J (2016). Inflammation and inflammatory cells in myocardial infarction and reperfusion injury: a double-edged sword. Clin Med Insights Cardiol.

[21] Couper KN, Blount DG, Riley EM (2008). IL-10: the master regulator of immunity to infection. J Immunol.

[22] Frangogiannis NG (2014). The inflammatory response in myocardial injury, repair, and remodelling. Nat Rev Cardiol.

[23] Zhou JH, Broussard SR, Strle K, Freund GG, Johnson RW, Dantzer R, Kelley KW (2001). IL-10 inhibits apoptosis of promyeloid cells by activating insulin receptor substrate-2 and phosphatidylinositol 3’-kinase. J Immunol.

[24] Zhu Y, Liu Z, Peng YP, Qiu YH (2017). Interleukin-10 inhibits neuroinflammation-mediated apoptosis of ventral mesencephalic neurons via JAK-STAT3 pathway. Int Immunopharmacol.

[25] Tao Z, Chen B, Tan X, Zhao Y, Wang L, Zhu T, Cao K, Yang Z, Kan YW, Su H (2011). Coexpression of VEGF and angiopoietin-1 promotes angiogenesis and cardiomyocyte proliferation reduces apoptosis in porcine myocardial infarction (MI) heart. Proc Natl Acad Sci U S A.

[26] Randi AM, Laffan MA (2017). Von Willebrand factor and angiogenesis: basic and applied issues. J Thromb Haemost.

[27] Orlic D, Kajstura J, Chimenti S, Jakoniuk I, Anderson SM, Li B, Pickel J, McKay R, Nadal-Ginard B, Bodine DM (2001). Bone marrow cells regenerate infarcted myocardium. Nature.

[28] Chen SL, Fang WW, Ye F, Liu YH, Qian J, Shan SJ, Zhang JJ, Chunhua RZ, Liao LM, Lin S, Sun JP (2004). Effect on left ventricular function of intracoronary transplantation of autologous bone marrow mesenchymal stem cell in patients with acute myocardial infarction. Am J Cardiol.

[29] Chavez A, Scheiman J, Vora S, Pruitt BW, Tuttle M, E PRI, Lin S, Kiani S, Guzman CD, Wiegand DJ (2015). Highly efficient Cas9-mediated transcriptional programming. Nat Methods.

[30] Nakajima M, Nito C, Sowa K, Suda S, Nishiyama Y, Nakamura-Takahashi A, Nitahara-Kasahara Y, Imagawa K, Hirato T, Ueda M (2017). Mesenchymal stem cells overexpressing Interleukin-10 promote neuroprotection in experimental acute ischemic stroke. Mol Ther Methods Clin Dev.

[31] Choi JJ, Yoo SA, Park SJ, Kang YJ, Kim WU, Oh IH, Cho CS (2008). Mesenchymal stem cells overexpressing interleukin-10 attenuate collagen-induced arthritis in mice. Clin Exp Immunol.

[32] Taga K, Chretien J, Cherney B, Diaz L, Brown M, Tosato G (1994). Interleukin-10 inhibits apoptotic cell death in infectious mononucleosis T cells. J Clin Invest.

[33] Cohen SB, Crawley JB, Kahan MC, Feldmann M, Foxwell BM (1997). Interleukin-10 rescues T cells from apoptotic cell death: association with an upregulation of Bcl-2. Immunology.

[34] Silvestre JS, Mallat Z, Duriez M, Tamarat R, Bureau MF, Scherman D, Duverger N, Branellec D, Tedgui A, Levy BI (2000). Antiangiogenic effect of interleukin-10 in ischemia-induced angiogenesis in mice hindlimb. Circ Res.

[35] Dace DS, Khan AA, Kelly J, Apte RS (2008). Interleukin-10 promotes pathological angiogenesis by regulating macrophage response to hypoxia during development. PLoS One.

[36] Kinzenbaw DA, Chu Y, Pena Silva RA, Didion SP, Faraci FM (2013). Interleukin-10 protects against aging-induced endothelial dysfunction. Physiol Rep.

