# Supplementary material for: Transplantation of CRISPRa system engineered IL10-overexpressing bone marrow-derived mesenchymal stem cells for the treatment of myocardial infarction in diabetic mice
Source: J Biol Eng. 2019 May 30;13:49. doi: 10.1186/s13036-019-0163-6 (PMC6543626; doi:10.1186/s13036-019-0163-6)
Supplement: Supplementary file 1 — Figure S1. (A) gRNAs (gIL10) were designed using the CRISPR.mit.edu platform, and 6 candidate gRNA sequence were selected according to the scores. (B) Activation of IL10 expression after transfected with different pCas9/gIL10. The activation of IL10 were examined by real-time PCR. The Guide#2 was selected for the further experiments. Figure S2. Representative confocal laser microscopic images of engrafted BM-MSCs at 4 weeks after transplantation. GFP positive cells indicated the alive BM-MSCs. Figure S3. The five sections of the representative Masson’s trichrome–stained myocardial sections in the control MI heart or hearts treated with BM-MSCs pCas9/gCtrl, pCas9/gIL10 or BM-MSCs pCas9/gIL10. Blue, scar tissue; red, viable myocardium. (PDF 233 kb) [file 13036_2019_163_MOESM1_ESM.pdf]

Supplementary materials

A

	Score	Sequence	
Guide #1	92	AACCACGTGTACCTAAGTCG	GGG
Guide #2	89	TAGGGATCTTCCAGTACTAA	CGG
Guide #3	82	CCTGCATTGGATCGGTATTC	GGG
Guide #4	76	GGTCATGGAATCTAAGTACA	AGG
Guide #5	72	GGAAATCATTAGTACTTGGA	TGG
Guide #6	71	AAGCTTACGAGTCGATGAAG	CGG

B

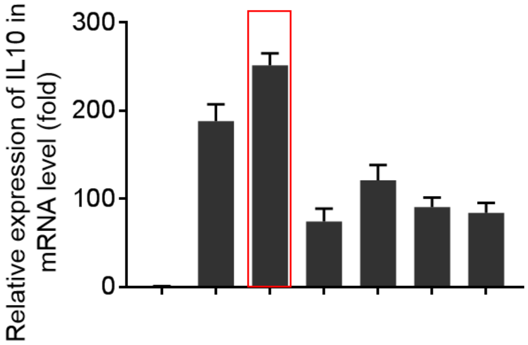

Figure S1. (A) gRNAs (gIL10) were designed using the CRISPR.mit.edu platform, and 6 candidate gRNA sequence were selected according to the scores. (B) Activation of IL10 expression after transfected with different pCas9/gIL10. The activation of IL10 were examined by real-time PCR. The Guide#2 was selected for the further experiments.

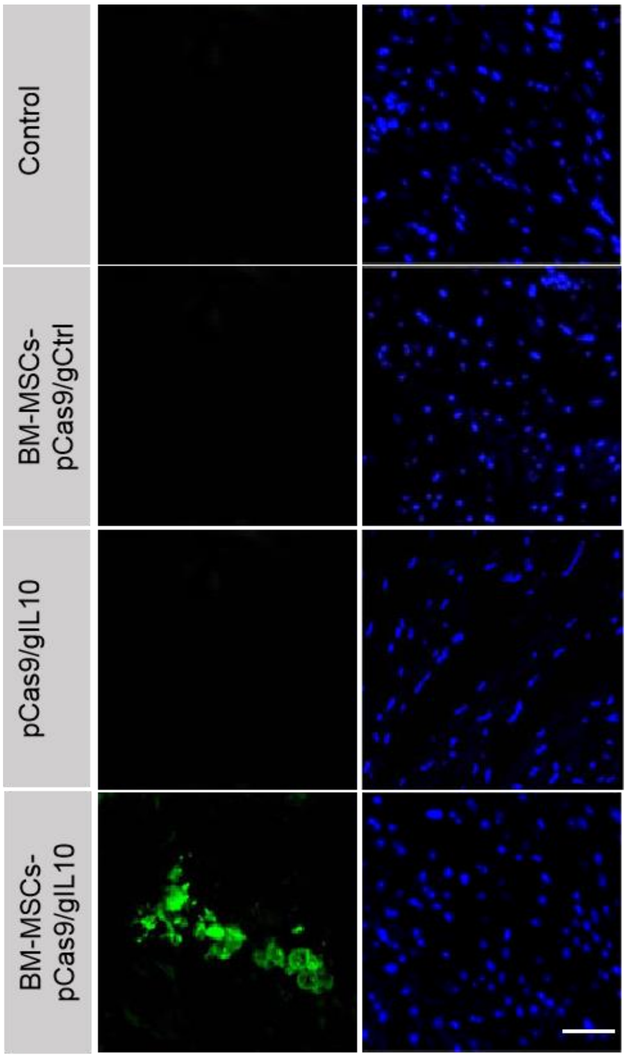

Figure S2. Representative confocal laser microscopic images of engrafted BM-MSCs at 4 weeks after transplantation. GFP positive cells indicated the alive BM-MSCs.

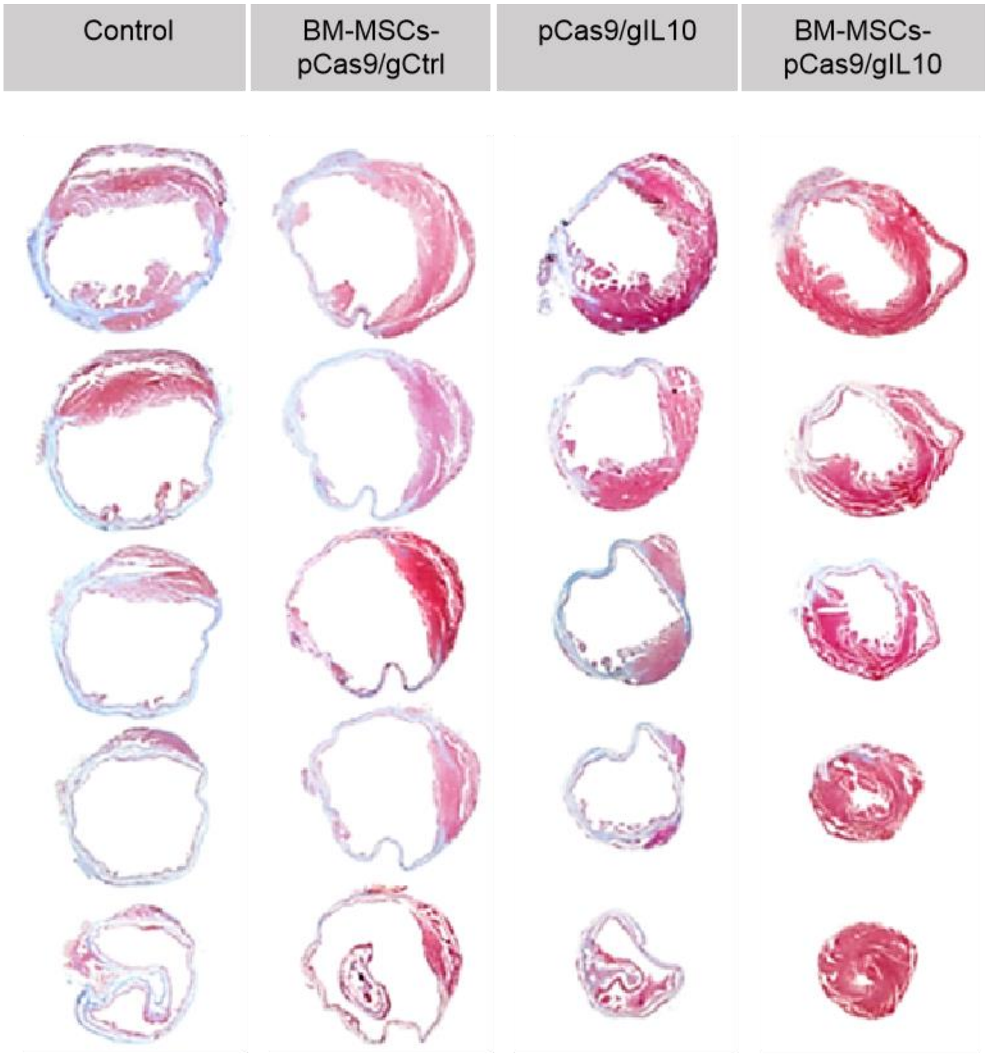

Figure S3. The five sections of the representative Masson's trichrome – stained myocardial sections in the control MI heart or hearts treated with BM-MSCs pCas9/gCtrl, pCas9/gIL10 or BM-MSCs pCas9/gIL10. Blue, scar tissue; red, viable myocardium.
